# Characterization of an Avian Influenza Virus H9N2 Strain Isolated from a Wild Bird in Southern China

**DOI:** 10.1128/genomeA.00600-14

**Published:** 2014-06-19

**Authors:** Qian Xu, Zhixun Xie, Liji Xie, Zhiqin Xie, Xianwen Deng, Jiabo Liu, Sisi Luo

**Affiliations:** a College of Animal Science and Technology, Guangxi University, Nanning, Guangxi Province, China; b Guangxi Key Laboratory of Animal Vaccines and Diagnostics, Guangxi Veterinary Research Institute, Nanning, Guangxi Province, China

## Abstract

We isolated an avian influenza virus H9N2 strain from a wild bird in the Guangxi Province of southern China in 2013 named A/turtledove/Guangxi/49B6/2013(H9N2) (GX49B6). We aimed to understand the genetic characters of the GX49B6 strain by analyzing the complete genome sequence. The results showed that our isolated strain has features of low pathogenic avian influenza viruses and viruses that infect humans. The discovery of the complete genome sequence of the GX49B6 strain may be helpful to further the understanding of the epidemiology and surveillance of avian influenza viruses in the field.

## GENOME ANNOUNCEMENT

Avian influenza A virus (AIV) is a single-strained negative-sense RNA virus belonging to the family *Orthomyxoviridae*. It causes a variety of infections in avians and mammals. H9N2 subtype AIVs are widespread in the world and are the most prevalent subtype of avian influenza viruses reported in China over the last decade (1). Although H9N2 is characterized as a low pathogenic avian influenza virus, occasional infections of humans (2–5) have caused great concerns. So far, H9N2 subtype AIVs are mainly isolated from domestic birds, however, wildfowl and shorebirds are the natural hosts of AIVs (6) and they facilitate the transmission of avian influenza (7). Guangxi is a province of major poultry industry and is contiguous to Vietnam where the avian influenza epidemic is complex. Enhancing the surveillance of H9N2 subtype AIVs among wild birds is very important.

In this study, we isolated an H9N2 strain named A/turtledove/Guangxi/49B6/2013(H9N2) (GX49B6) from a wild bird in the Guangxi province. We amplified eight genes of the GX49B6 strain by RT-PCR using the universal primers of the influenza A virus (8, 9).

The complete genome of GX49B6 strain consists of PB2, PB1, PA, HA, NP, NA, M, and NS segments. The full lengths of the segments are 2,341 nucleotides (nt), 2,341 nt, 2,233 nt, 1,742 nt, 1,565 nt, 1,459 nt, 1,027 nt, and 890 nt, respectively. The amino acid lengths of the proteins encoded by the eight genes as follows: PB2, 759 aa; PB1, 757 aa; PA, 716 aa; HA, 560 aa; NP, 498 aa; NA, 466 aa; M2, 97 aa; M1, 252 aa; NS2, 121 aa; NS1, 217 aa. The amino acid residue at the cleavage site (335-341) of the HA molecule is RSSR↓GLF, without multiple consecutive basic amino acids, which is characteristic of low pathogenic AIVs. The presence of 158 Glu, 627 Glu, 701Asp in the amino acid sequence of the PB2 protein, 436 Tyr in the PB1 protein, and 515 Thr in the PA protein, respectively, also provides evidence of low pathogenicity (10, 11). The GX49B6 strain has L226 and G228 (according to H3 numbering) at the receptor-binding site in the HA protein, which suggests that the GX49B6 strain might have the ability to bind a sialic acid-2,6-NeuAcGal linkage and might have the potential to infect humans (4, 12).

Sequence analysis revealed that the nucleotide sequences of the HA and NA genes of the GX49B6 strain both belong to the Eurasian lineage. The HA gene shared 96.3% nucleotide homology with the isolate A/Chicken/Guangxi/55/2005(H9N2) that is thought to be a representative strain in China since 2007 (13). The PB2 and PB1 genes both share a high homology (≥97%) with genes of the H7N9 strains isolated from infected humans in Guangdong and Anhui provinces, respectively. They are also similar to genes of some strains isolated from Vietnam (≥98%).

The data demonstrate that wild birds are involved in the transmission and evolution of AIVs. Our results may be helpful in epidemiological studies on H9N2 subtype AIVs.

### Nucleotide sequence accession numbers.

The virus genome sequence of the A/turtledove/Guangxi/49B6/2013(H9N2) strain was deposited in the DDBJ/EMBL/GenBank database under accession numbers KJ725009 through KJ725016.
